# How Gut Microbes Transform Dietary Phytonutrients: Enzymatic Pathways and Human Metabolite Exposure

**DOI:** 10.3390/molecules31162895

**Published:** 2026-08-19

**Authors:** Roko Šantić, Marko Kumrić, Marino Vilović, Nikola Pavlović, Doris Rušić, Josipa Bukić, Joško Božić

**Affiliations:** 1 Department of Pathophysiology, University of Split School of Medicine, 21000 Split, Croatia; roko.santic@mefst.hr (R.Š.); marko.kumric@mefst.hr (M.K.); marino.vilovic@mefst.hr (M.V.); nikola.pavlovic@mefst.hr (N.P.); 2 Laboratory for Cardiometabolic Research, University of Split School of Medicine, Soltanska 2A, 21000 Split, Croatia; 3 Department of Pharmacy, University of Split School of Medicine, Soltanska 2A, 21000 Split, Croatia; doris.rusic@mefst.hr (D.R.); josipa.bukic@mefst.hr (J.B.); 4 Department of Laboratory Medicine and Pharmacy, Faculty of Medicine, Josip Juraj Strossmayer University of Osijek, Josipa Huttlera 4, 31000 Osijek, Croatia

**Keywords:** gut microbiota, dietary phytonutrients, polyphenols, microbial enzymes, biotransformation, S-equol, urolithins, enterolignans, isothiocyanates, phenyl-γ-valerolactones

## Abstract

Compounds identified in plant foods are often not the species that reach human tissues. Glycosides, tannins and incompletely absorbed aglycones enter the colon, where gut microorganisms hydrolyse, reduce, cleave, demethylate or dehydroxylate them. This critical narrative review examines pathways resolved beyond taxonomic association: daidzein-to-S-equol conversion; cooperative urolithin and enterolignan formation; flavone reduction and C-ring cleavage; stereoselective catechin metabolism; glucosinolate activation; and transformations of resveratrol and hydroxycinnamate-derived metabolites. We distinguish enzyme and genetic evidence from findings in isolates, consortia, faecal communities, gnotobiotic models and human studies. Detecting a taxon or catalytic gene does not establish in vivo pathway flux. Substrate release, transcription, cofactor supply, cross-feeding, product consumption, absorption and host conjugation shape the metabolites detected in plasma and urine. Human studies identify distinct S-equol and urolithin phenotypes and show substantial exposure to phenyl-γ-valerolactones and smaller phenolic acids, although several high-flux reactions remain enzymatically unresolved. Biological relevance is most credible when tested with circulating conjugates at concentrations attained after food intake. Linking reaction-level microbiology to measured exposure may explain interindividual responses to phytonutrient-rich foods. We conclude that microbiota-dependent exposure should be claimed only when reaction-level evidence is connected to precursor-specific human metabolites. This framework separates established conversion from unresolved spontaneous and host processing and specifies the evidence needed to explain individual responses.

## 1. Introduction

Plant-food composition differs from the exposure produced after ingestion. Dietary polyphenols occur as glycosides, esters, oligomers and tannins whose size, polarity and food-matrix associations affect upper-gastrointestinal absorption. Absorbed compounds are often converted into sulfates, glucuronides or methylated derivatives. The distal fraction instead encounters anaerobic microorganisms that hydrolyse glycosides, reduce double bonds, open heterocycles and remove methoxy or hydroxyl groups [1,2,3]. Their products may be absorbed, retained locally, consumed by other organisms or further modified by the host.

The familiar claim that “the microbiome increases polyphenol bioavailability” is too broad. Conversion may create a product with a new target profile, as with S-equol from daidzein. It may instead remove a relevant functional group, divert a precursor or detoxify a compound that inhibits neighbouring bacteria. The word microbiome also hides the level of evidence. A 16S rRNA–metabolite correlation identifies no reaction; purified-enzyme activity does not show colonic expression. Isolate conversion does not establish absorption, and a circulating metabolite need not mediate a clinical effect. Collapsing these levels turns a plausible pathway into an apparently settled causal chain.

Comparative genomics, substrate-responsive transcriptomics, anaerobic protein biochemistry and genetic reconstruction have assigned reactions previously seen only in faecal incubations [4,5]. They include flavone reduction, a distributed lignan pathway, regioselective urolithin dehydroxylation, enantiocomplementary catechin enzymes and multicomponent glucosinolate activation. Recent reviews cover much of this enzymology [6] and the broader chemistry of food bioactives [7]. Here, we link assigned reactions to native metabolites measured after human intake while keeping unresolved steps explicit.

We follow compounds from dietary precursor to microbial product and, where evidence permits, to systemic exposure. The review covers common food phytonutrients and includes a pathway only when at least one organism-level transformation has been chemically demonstrated. Central sections prioritise gene- or enzyme-assigned reactions, assess the strength of each assignment and ask whether the product has been detected after human intake. High-exposure pathways with incomplete enzymology are discussed separately rather than presented as fully resolved. Health evidence is treated conservatively: direct supplementation with a microbial product is distinguished from the consumption of its precursor, and experiments using unconjugated aglycones are not equated with the conjugated species and concentrations found in human plasma.

## 2. Literature Search and Scope

This is a critical narrative review, not a systematic review or meta-analysis. PubMed was searched from inception to 20 July 2026 by combining gut or intestinal microorganisms with named phytonutrients, metabolites and mechanistic terms, including enzyme, gene, reductase, hydrolase, dehydroxylase, catabolism and conversion. Separate searches covered daidzein/S-equol, ellagitannins/urolithins, plant lignans/enterolignans, flavones and flavonols, catechin and epicatechin, glucosinolates/isothiocyanates, resveratrol, hydroxycinnamates, phenyl-γ-valerolactones, anthocyanin-derived phenolic acids, curcuminoids and human metabolite exposure. Backward and forward citation tracking recovered older biochemical studies, later functional work and chemistry papers poorly indexed by nutritional terms. Bibliographic metadata and DOI–title identity were checked for every cited article. Reported or deposited protein sequences were compared when independently named loci appeared to encode the same reaction.

Eligible evidence comprised the conversion of a defined substrate to a chemically identified product by a purified enzyme, recombinant system or isolate; genetic gain or loss of function; defined consortia; ex vivo human faecal conversion; microbiologically controlled animal experiments; and human intervention or pharmacokinetic studies measuring the relevant precursor and product. Focused reviews guided citation tracking but did not replace primary evidence for mechanistic or human-exposure claims.

Studies were excluded from the evidentiary core if they reported only community-composition changes after a plant extract, relied solely on in silico enzyme annotation, used an incompletely characterised botanical mixture without identifying the relevant substrate, or inferred a health mechanism from generic antioxidant assays. Short-chain fatty acids, bile acids, trimethylamine, drugs and non-dietary xenobiotics were outside the scope unless there was a need to explain an enzyme family or method. In vitro biological assays were considered physiologically informative only when chemical form and concentration were compatible with measured intestinal or systemic exposure. Because terminology and taxonomy have changed, strain identifiers and names from the primary experiment are retained where needed to avoid ambiguity.

The evidence vocabulary is deliberately asymmetric. “Catalyses” is reserved for reactions demonstrated with a purified or recombinantly expressed enzyme. “Is required” denotes genetic loss of function in the relevant organism or system, whereas “is sufficient” denotes gain of activity after heterologous reconstruction. “Produces” indicates a chemically verified isolate or community phenotype without necessarily identifying its enzyme. “Associated with” applies to observational human or community-level relationships. These terms prevent experimentally distinct claims from collapsing into one pathway. Because this was a critical narrative review rather than a systematic review, PRISMA 2020 was not applied, and stage-specific screening counts were neither prospectively recorded nor retrospectively estimated. To improve transparency, Supplementary Figure S1 summarises the search process, eligibility criteria, evidence classification and synthesis workflow.

## 3. Dietary Precursor Delivery to the Colon

Neither the quantity nor the chemical form of a phytochemical entering the colon can be inferred by applying a universal “poorly absorbed” fraction to the ingested dose. Proximal handling varies with molecular size, glycosylation, stereochemistry, solubility, food-matrix binding and processing. Accessible glycosides may be hydrolysed at the brush border or in enterocytes, then absorbed and conjugated. Polymeric procyanidins, ellagitannins and phenolics trapped in an insoluble matrix are less available to small-intestinal transport. The colonic inoculum is therefore meal- and compound-specific, not merely the unchanged dietary precursor. Extra-virgin olive oil illustrates the same principle: cultivar, ripening stage, processing, storage and digestion reshape the phenolic profile to which the intestine is ultimately exposed [8].

Formulation studies illustrate this principle but do not establish in vivo colonic delivery. Eggplant-peel anthocyanins bound lactoferrin-derived peptides through van der Waals interactions and hydrogen bonding [9]. Separately, a whey-protein/pectin matrix gave the greatest carotenoid release among five microencapsulated sea-buckthorn formulations during simulated digestion [10]. Neither study quantifies microbial access after eating a natural food. Both show why matrix binding and release should be measured rather than inferred from total phytochemical content.

These formulation examples become relevant to lower-gut kinetics when matrix structure persists through digestion. Mechanical disruption and food pre-fermentation can expose intracellular or bound substrates before colonic entry, whereas intact cell walls and protein–polysaccharide carriers may alter release and redistribute substrate delivery over time. In a digestion–faecal fermentation model, particle size altered flaxseed lignan bioaccessibility, while pre-fermentation increased subsequent secoisolariciresinol release and enterolignan conversion [11]. Equal total phytochemical doses can therefore generate different concentration–time profiles in the colon, altering which organisms gain first access and whether intermediates accumulate or are immediately cross-fed. Lower-gut studies should report particle size, thermal and mechanical processing, carrier composition, predigestion conditions and time-resolved substrate release.

Human ileostomy studies offer the most direct estimate of material escaping the small intestine. After green tea, approximately 70% of administered flavan-3-ols appeared in ileal fluid over 24 h: about one-third as parent compounds and another 37% as 23 identified or putatively identified metabolites [12]. Thus, “unabsorbed polyphenol” is a mixed substrate pool shaped by digestion and host metabolism. Chemical form also mattered in apple studies. Recovery of individual phenolics after cloudy apple juice ranged from undetectable to about one-third of intake [13]. By contrast, approximately 90% of procyanidins reached the effluent, although their mean degree of polymerisation fell from about 5.7 in juice to 3.4 in effluent [14]. Substantial distal delivery can therefore coexist with pre-colonic modification. Raspberry ileostomy sampling similarly detected anthocyanins and ellagitannin-related material beyond the small intestine [15].

These measurements describe upper-gastrointestinal output, not a microbiologically available dose. Ileal effluent contains parent molecules, hydrolysis products, host conjugates and perhaps compounds returned to the lumen by enterocyte or biliary secretion. It does not reveal how tightly the material remains matrix-bound or how quickly it becomes accessible in the colon. Ileostomy subjects also lack a functioning colon and may differ from colon-intact volunteers in transit and enterohepatic cycling. The conclusion must therefore be narrower than the claim that most polyphenols “reach the colon intact”. High-molecular-weight and matrix-bound fractions often arrive in substantial amounts, whereas smaller molecules undergo variable proximal absorption. Microbes encounter a pre-processed mixture, not merely the labelled dietary compound.

## 4. Experimentally Resolved Microbial Pathways

The pathways below are organised by reaction and followed only as far as enzyme biochemistry, genetic perturbation, heterologous reconstruction or a chemically defined isolate or consortium allows.

### 4.1. Soy Isoflavones and S-Equol: A Stereochemically Constrained Microbial Bioactivation

Soy provides daidzein mainly as daidzin and related glycosides. Deglycosylation releases the aglycone, yet chemical form alters proximal exposure: in a crossover study, daidzin produced a substantially greater daidzein area under the curve than an aglycone-equivalent dose [16]. Equol formation is a separate, strictly anaerobic pathway that converts achiral daidzein into (3S)-equol, the human bacterial enantiomer and a preferential oestrogen-receptor-β ligand [17]. A standardised daidzein challenge can reveal this catalytic phenotype; soy intake alone cannot.

Daidzein reductase (DZNR) catalyses the reduction in daidzein to (R)-dihydrodaidzein (DHD); dihydrodaidzein racemase (DDRC) catalyses racemisation to S-DHD; dihydrodaidzein reductase (DHDR) catalyses its reduction to tetrahydrodaidzein; and tetrahydrodaidzein reductase (THDR) catalyses the final reduction to yield S-equol. Gene-library and recombinant-enzyme studies in Slackia sp. NATTS and Lactococcus sp. 20–92 established this order and the racemisation [18,19]. In Slackia isoflavoniconvertens, an induced eight-gene region was linked to daidzein and genistein conversion, and recombinant proteins reproduced the three reductions [20]. Homologous systems vary: Eggerthella sp. YY7918 enzymes showed an atypical dismutase-like terminal reaction, while its octameric DZNR contains FMN, FAD and iron–sulfur centres consistent with enantioselective first reduction [21,22].

A four-gene segment from Adlercreutzia equolifaciens DSM 19450ᵀ is sufficient in Escherichia coli to produce equol but performed poorly or not at all in two lactic-acid bacteria [23]. The activity of the catalytic genes is therefore host-context dependent. A non-producing A. equolifaciens strain lacks much of the operon [24], and omitting DDRC markedly reduced output in an engineered system [25]. Among 47,894 representative genomes, complete clusters appeared in only 18 genomes and varied within named species [26]. Daidzein induces coordinated transcription in DSM 19450ᵀ [27], yet some faecal samples carry all four PCR targets without producing detectable equol [25]. Cluster integrity and expression are more informative than genus abundance.

The catalytic quartet operates within a wider redox system. Conserved neighbouring genes encode predicted electron-transfer proteins and hydrogenase-maturation functions, while the cofactor-rich YY7918 DZNR illustrates the first reduction’s electron demand [22,26]. Unequal yields after transfer between hosts may reflect promoter recognition, flavin and iron–sulfur assembly, reductant supply or substrate transport. No native targeted-mutant series has tested all four genes in a strictly anaerobic producer. For most steps, recombinant sufficiency therefore remains stronger than native-host necessity.

Whole-organism studies show that the sequence is not a recombinant-assay artefact. S. isoflavoniconvertens HE8 produced equol from daidzein through DHD in culture [28] and produced equol and 5-hydroxyequol in monoassociated gnotobiotic rats [29]. The steps may also be divided between organisms: a two-member culture paired daidzein-to-DHD and DHD-to-equol isolates. One member, however, came from bovine rumen, and the pairing was not demonstrated in humans [30].

Faecal incubations add ecological realism but lose organism-level attribution. Six of 17 donor communities produced equol from daidzein, but equol later declined in several cultures. Pre-fermenting a soy beverage with a daidzin-deglycosylating bifidobacterium increased recovery in selected systems [31]. Precursor release can therefore alter pathway input, while net faecal accumulation reflects both production and subsequent loss.

Human challenge studies establish frequency and persistence, not mechanism. Producer prevalence differed between Korean American and Caucasian American participants; repeat classification one to three years later was highly, though not perfectly, concordant [32,33]. After a defined soy challenge, a urinary log equol:daidzein ratio separates producers better than a single absolute concentration [34]. Ten weeks of high-soy intake changed faecal β-glucosidase activity but generally did not convert non-producers [35]. Classification still depends on dose, threshold and sampling interval, and urinary equol integrates formation, absorption and host conjugation.

The phenotype is quantitative, not merely binary. In a controlled dietary study, equol excretion spanned more than two orders of magnitude; associations with habitual macronutrient intake were exploratory, not causal [36]. A participant near the threshold may change category without gaining or losing the pathway. Cross-cohort comparisons should report the administered daidzein equivalent, equol:daidzein ratio, collection interval and chiral analytical method.

Evidence that producer status modifies clinical benefit is weaker. A pilot trial of menopausal hot flushes suggested larger dose-related changes among producers, but the interaction was inconclusive [37]. A later skin pilot found no significant group difference in its primary crow’s feet outcome, although exploratory measures tracked urinary equol in producers [38]. The pathway creates a distinct exposure. Attributing benefit to endogenous production still requires trials measuring microbial activity, equol kinetics and prespecified outcomes in the same participants.

### 4.2. Ellagitannins and Urolithins: Successive Dehydroxylations Create Distinct Metabolic Phenotypes

Ellagitannins in pomegranate, walnuts and berries release ellagic acid, not preformed urolithins. Human faecal cultures first showed that ellagic acid and related tannins yield urolithin A [39], but isolate work has resolved only part of the sequence. Gordonibacter urolithinfaciens and Gordonibacter pamelaeae produced urolithin C from ellagic acid through urolithins M5 and M6, without forming less-hydroxylated terminal products [40]. Ellagibacter isourolithinifaciens produced isourolithin A [41]. These are organism-level transformations; proteins responsible for lactone opening and several early dehydroxylations remain unknown.

The terminal route to urolithin A is clearer. Selected Enterocloster strains produce urolithin A from urolithin C; an inducible ucdCFO operon encodes a three-subunit molybdopterin–cytosine–dinucleotide complex with FAD- and iron–sulfur-containing electron-transfer components. Induced cells produced urolithin A from urolithin C, and heterologous ucdCFO expression is sufficient to confer activity on an inactive host [42]. Without a reported native Enterocloster deletion, heterologous sufficiency is stronger than native-host necessity.

Anaerobic molybdoenzymes are difficult to reconstitute because their catalytic, iron–sulfur and flavin subunits may not form a soluble purified complex. For Ucd, convergent transcriptomics, proteomics, substrate induction and heterologous crude-lysate activity support assignment despite the absence of a fully purified complex. This is an enzyme-complex assignment, not single-protein catalysis. It applies to the 9-hydroxyl group of suitable urolithin intermediates and does not resolve ellagic-acid lactone opening or decarboxylation.

A second operon, uxdFOC, explains 10-position dehydroxylation by E. asparagiformis, E. citroniae and E. pacaense, an activity absent from E. bolteae. Urolithin M6 released by Gordonibacter gave different product profiles with individual Enterocloster strains. Heterologous uxdFOC expression is sufficient to confer 10-position dehydroxylation [43]. This demonstrates cross-feeding in the reconstructed terminal route, not a universal requirement for those partners.

Defined-community and faecal experiments show where genomic potential can fail. Selected consortia transferred A-like or B-like product profiles to non-producing rats given ellagic acid [44]. In human faecal slurries challenged with urolithin C, urolithin A formation tracked full-length ucd transcription, although the relevant organisms and DNA were also present in non-converting samples [42]. Induction and physiological state therefore add information beyond taxonomic detection.

Ex vivo challenges also localise a non-producer’s deficit. Failure to form urolithin A from ellagic acid may arise upstream, before urolithin C formation. Failure after direct urolithin-C challenge instead implicates the terminal branch or its expression conditions. Substrate-resolved challenges are more informative than one endpoint after pomegranate intake, especially when communities consume intermediates or form alternative regioisomers.

Human phenotyping distinguishes metabotype A, dominated by urolithin A; metabotype B, which also includes isourolithin A and/or urolithin B; and metabotype 0, with no detected terminal urolithins. Their distribution differed cross-sectionally among 839 participants aged 5–90 years [45]. This is an age-associated population pattern, not evidence of within-person switching. After pomegranate juice, urolithin conjugates appeared later than ellagic acid, persisted in urine for up to 48 h and varied markedly between individuals [46]. In a pomegranate-extract intervention, faecal urolithin A correlated with changes in bile-acid and cholesterol metabolism, without proving mediation [47].

Matrix and formulation may alter timing without abolishing the phenotype. Juice, liquid extract and powdered pomegranate products produced broadly similar urinary urolithin-A-glucuronide output in a small sequential intervention, although powder delayed ellagic-acid appearance [48]. This supports formulation-sensitive precursor release, not bioequivalence among commercial ellagitannin preparations.

Tissue detection also demands restraint. Urolithin A glucuronide was found in prostate tissue after walnut and pomegranate intake, but proliferation markers did not change [49]. Direct urolithin A dosing produces more uniform exposure than ellagitannin-rich foods because it bypasses substrate release and microbial conversion [50]. Terminal dehydroxylation is unusually well resolved; early enzymes, person-level gene-to-flux relationships and the exposure needed for biological effects are not.

### 4.3. Plant Lignans and Enterolignans: A Distributed Pathway Across Low-Abundance Gut Bacteria

Plant lignans enter microbial metabolism through several substrates. Flaxseed is rich in secoisolariciresinol diglucoside (SDG); pinoresinol, lariciresinol and matairesinol occur across seeds, grains and vegetables. Glycosides require hydrolysis, and pinoresinol undergoes two benzyl-ether reductions before the routes converge on enterodiol and enterolactone. Early isolate and faecal-community studies showed that SDG conversion is sequential and distributed across phylogenetically distinct bacteria [51,52,53].

The entry reaction is assigned to the benzyl-ether reductase Ber of Eggerthella lenta. Pinoresinol metabolism varies by strain, not species phylogeny. A locus containing ber and its putative regulator was present in metabolising strains and was induced by pinoresinol; the heterologous expression of the locus is sufficient in Escherichia coli to convert pinoresinol through lariciresinol to secoisolariciresinol [54]. Active-site substitutions changed product formation. Together, natural variation, induction and heterologous activity make Ber the best-resolved lignan catalyst.

Three downstream organisms complete the route, with unequal mechanistic certainty. Blautia producta produces didemethylsecoisolariciresinol from secoisolariciresinol; an induced glm locus tracks the reaction but has not been reconstituted. Gordonibacter pamelaeae produces enterodiol, yet its induced molybdopterin-enzyme candidate cldh also lacks purified or heterologous confirmation. By contrast, Lactonifactor longoviformis produces enterolactone from enterodiol, and the heterologous expression of the induced dehydrogenase edl is sufficient to confer activity [54]. Glm and Cldh therefore remain candidate catalysts, unlike Ber and Edl.

The missing evidence is reaction-specific. The failed heterologous expression of Glm leaves open whether it needs a partner or unusual methyl acceptor. For Cldh, strong induction and restriction to metabolising Coriobacteriia nominate a molybdopterin oxidoreductase but do not show that the protein forms enterodiol. Purifying complete cofactor-containing modules or combining native deletions with chemical rescue would establish catalysis.

**Figure 1:**
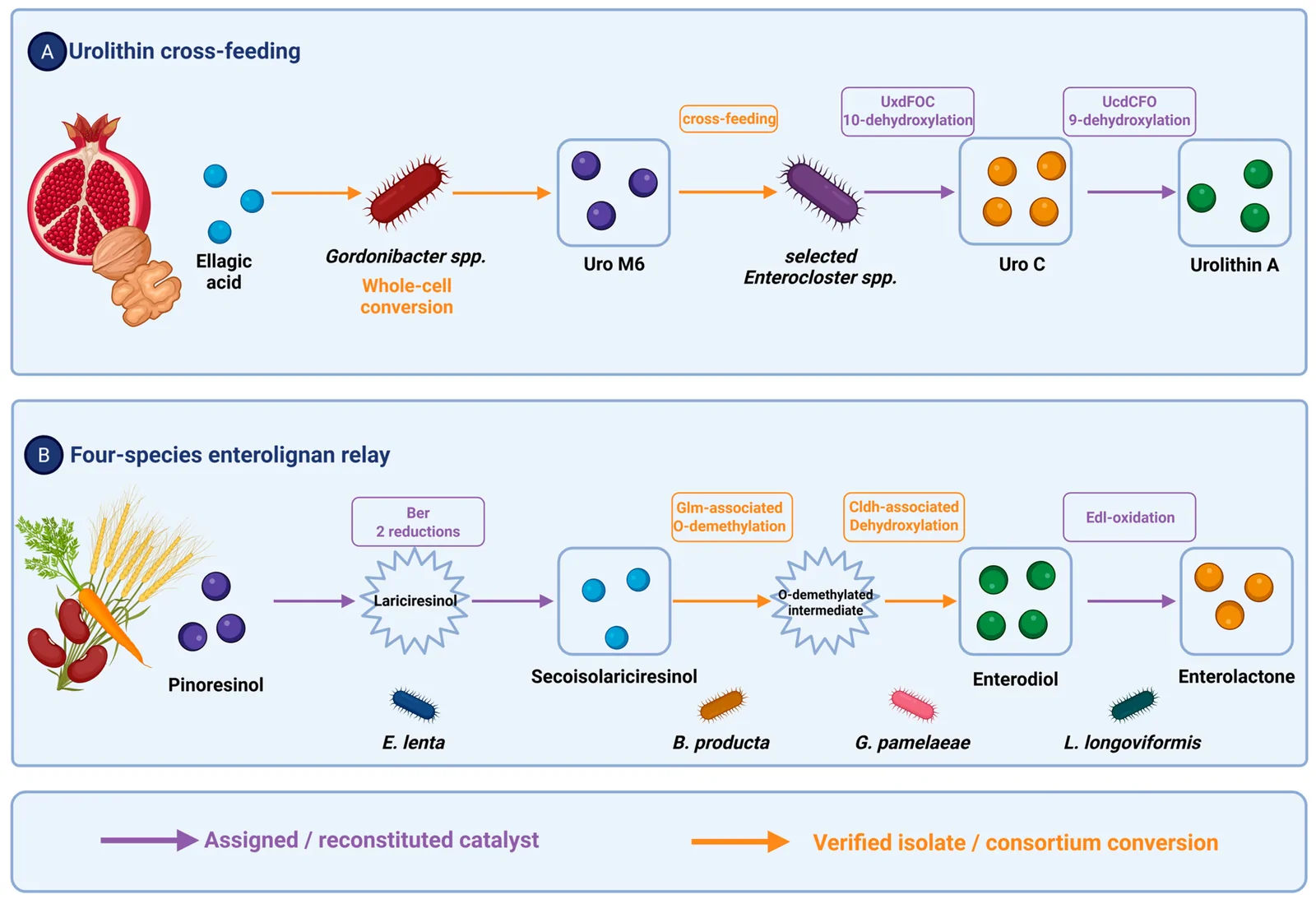
Microbial division of labour in urolithin and enterolignan formation. (**A**) In a reconstructed cross-feeding route, Gordonibacter spp. sequentially produce urolithins M5 and M6 from ellagic acid and release M6 into the extracellular medium. Selected Enterocloster species carrying functional Uxd and Ucd complexes then perform the terminal transformations: UxdFOC catalyses 10-dehydroxylation of urolithin M6 to urolithin C, whereas UcdCFO catalyses 9-dehydroxylation of urolithin C to urolithin A. The alternative terminal route via urolithin M7 and the formation of urolithin C by Gordonibacter spp. are not shown. (**B**) A representative four-species enterolignan relay produces enterolactone from pinoresinol. Eggerthella lenta Ber catalyses two successive benzyl-ether reductions, yielding lariciresinol and secoisolariciresinol. Blautia producta produces didemethylsecoisolariciresinol (dmSECO) from secoisolariciresinol, and Gordonibacter pamelaeae produces enterodiol from dmSECO; Glm and Cldh are substrate-induced candidate catalysts for these reactions but have not been independently reconstituted. Lactonifactor longoviformis produces enterolactone from enterodiol, and heterologous Edl expression is sufficient to confer this activity. Purple arrows indicate reactions functionally assigned through heterologous expression; solid orange arrows indicate verified whole-cell or consortium conversions without independent catalyst-level confirmation; the dashed orange arrow indicates observed metabolite release and cross-feeding. The schemes depict selected experimentally supported routes and do not imply a unique or universal human consortium. Created with BioRender.

Combining E. lenta, B. producta, G. pamelaeae and L. longoviformis reconstituted five transformations from pinoresinol to enterolactone. An earlier four-member consortium produced enterolactone in gnotobiotic rats [55], and communities containing ber-positive rather than ber-negative E. lenta differed in enterodiol output in gnotobiotic mice [54]. These experiments reproduce community-level conversion under gnotobiotic conditions; they do not establish a unique human consortium. Further intermediates detected in faecal cultures and after flaxseed intake indicate branching beyond a single five-arrow route [56]. Figure 1 compares this four-species enterolignan relay with the reconstructed urolithin cross-feeding route and distinguishes catalyst-level assignments from conversions supported only at the isolate or consortium level.

Low enterolactone concentrations persisted in some germ-free or *ber*-negative conditions in the later mouse experiments [54]. Background dietary lignans, alternative entry substrates and analytical carry-over must therefore be considered before one administered precursor is treated as the sole source. The decisive comparison is the substrate-dependent difference between matched communities, and not detection of the terminal molecule alone.

Human exposure begins with matrix release. In a crossover study, enterolignan bioavailability from whole or crushed flaxseed was only 28% or 43%, respectively, of that from milled seed [57]. After a single SDG dose, enterodiol and enterolactone appeared at about 8–10 h, with median peaks near 15 and 20 h, consistent with sequential colonic formation [57]. Longer interventions show marked differences between low and high excreters. In one trial, microbiome features tracked urinary enterolactone, but transcriptomic changes met the prespecified threshold in exfoliated cells, not mucosal biopsies [58].

Circulating enterodiol and enterolactone are mostly conjugated, so urinary recovery reflects both microbial formation and host clearance. Ordered kinetics support a colonic sequence more strongly than a single endpoint urine sample, but they do not identify the limiting consortium member. Measuring intermediate lignans alongside ber, glm, cldh and edl transcripts could distinguish slow pathway entry from terminal-step failure.

Community disruption offers supportive, not definitive, evidence. Prior oral antibiotic use was associated with lower serum enterolactone in a large cohort, and the association persisted for months [59]. Diet, antibiotic indication and intestinal physiology may confound it. The biochemical conclusion is firmer: common plant lignans generate systemically available enterolignans through a cooperative, strain-sensitive pathway. The central demethylation and dehydroxylation catalysts, and any health benefit attributable to this conversion, remain unresolved.

### 4.4. Reduction and Ring Cleavage of Flavones and Flavonols

In the resolved Flavonifractor plautii route, the flavone or flavonol C2=C3 bond is reduced before heterocycle cleavage. The FMN-containing flavone reductase FLR catalyses apigenin reduction to naringenin and also accepts luteolin, kaempferol and quercetin, but not the heavily tested methoxylated flavones, isoflavones or anthocyanidins [60]. Earlier whole-cell studies showed that Eubacterium ramulus and Clostridium orbiscindens degrade selected flavones and flavonols into smaller aromatic products; enzyme work now defines one route behind that broader phenotype [61,62].

Whole-cell products reveal substrate-dependent branches. E. ramulus produced 3,4-dihydroxyphenylacetic and hydroxyphenylpropionic acids from quercetin and luteolin through reduced or ring-opened intermediates, whereas C. orbiscindens produced distinct product profiles from apigenin, luteolin and quercetin [61,62]. These studies establish pathway breadth but cannot assign each intermediate’s disappearance to one catalyst. FLR resolves the entry reaction for some conversions; it does not replace the isolate evidence.

Purified FLR needed an auxiliary flavin reductase to supply reduced FMN, leaving its physiological electron donor unknown. The flr homologue in Clostridium ljungdahlii is required for the consumption of several substrates under the tested conditions: deletion abolished consumption and complementation restored it [60]. This establishes genetic necessity in that host, not native-gene necessity in F. plautii. Experiments in gnotobiotic rats with quercetin-3-glucoside confirm in vivo bacterial degradation while separating glycoside hydrolysis from aglycone catabolism [63].

Reduction alone does not open the ring. The oxygen-sensitive Fcr of E. ramulus catalyses the stereoselective benzyl-ether cleavage of selected flavanones and flavanonols to a dihydrochalcone [64]. Phloretin hydrolase catalyses the cleavage of the inter-ring carbon–carbon bond, yielding phloroglucinol and 3-(4-hydroxyphenyl)propionic acid [65]. The purified chalcone isomerase catalyses the ring contraction of (+)-(2R,3R)-taxifolin to alphitonin [66]. Closely related substrates thus follow different routes.

Under the tested conditions, Fcr does not accept unsaturated flavones and flavonols, catechin or epicatechin. Continuity between FLR and Fcr therefore requires the correct reduced stereoisomer, compatible cellular localisation and access to another organism when the enzymes occur in different strains. Alphitonin formation preserves a different part of the carbon skeleton than direct dihydrochalcone cleavage. Collapsing both alternatives into “C-ring opening” erases demonstrated stereochemistry.

Community context matters even when the catalyst is known. Starch use by Bacteroides thetaiotaomicron promoted quercetin degradation and butyrate production by E. ramulus, indicating carbohydrate cross-feeding rather than the transfer of the flavonoid reaction [67]. The apigenin sequence—double-bond reduction, reductive ring opening and phloretin hydrolysis—is chemically coherent, but its quantitative continuity after human intake is unproven. An FLR-like sequence alone does not establish substrate delivery, expression or compatible stereochemistry, much less downstream systemic exposure.

The bridge to human exposure is clearest for the flavonol quercetin rather than for every substrate accepted by FLR. After a quercetin-3-rutinoside challenge, colon-intact participants excreted 3-hydroxyphenylacetic, 3-methoxy-4-hydroxyphenylacetic and 3,4-dihydroxyphenylacetic acids at approximately 36%, 8% and 5% of the administered dose, respectively, whereas participants without a colon formed only traces [68]. These findings show that colonic C-ring catabolism can generate phenylacetic acids that are absorbed and systemically recovered. They do not demonstrate that FLR, Fcr and phloretin hydrolase carry the complete sequence in humans, because urinary products integrate multiple organisms, further microbial reactions and host conjugation.

### 4.5. Stereoselective Metabolism of Catechin and Epicatechin

Catechin and epicatechin cannot be assigned to the flavone pathway simply because both are flavonoids. Their saturated C-ring has two stereocentres, and isolate work showed stereoselective ring fission and dehydroxylation [69]. In Eggerthella lenta, catechin benzyl-ether reductase Cber catalyses the C-ring opening of (+)-catechin and (−)-epicatechin, producing oppositely configured diarylpropan-2-ol intermediates. Heterologous Cber expression is sufficient for both conversions [70].

Configuration governs the next reaction. Molybdenum-dependent catechin dehydroxylase Cadh catalyses the dehydroxylation of the R intermediate from (+)-catechin, whereas independently induced eCadh catalyses the dehydroxylation of the S intermediate from (−)-epicatechin. The reconstruction of regulatory, catalytic and accessory genes established their enantiocomplementarity [70].

Cadh and eCadh are multisubunit systems that combine bis-molybdopterin-guanine-dinucleotide catalytic components with functions for cofactor insertion and electron transfer. Their opposite substrate preferences were demonstrated in reconstructed enzyme systems, not inferred from taxonomic correlation. Later, an E. lenta Vadh and a related Gordonibacter Vadh showed narrower substrate ranges in whole-cell assays than in purified-protein assays, consistent with transport or differential induction [70]. Catalytic-subunit abundance alone therefore cannot predict chiral product output.

Cocultures of F. plautii and E. lenta produced enantiomeric hydroxyvaleric acids from (+)-catechin and (−)-epicatechin [70]. This links assigned early reactions to five-carbon products, but does not establish that Cber, Cadh and eCadh alone synthesise phenyl-γ-valerolactones. Enzymes for A-ring carbon loss, several oxidations and lactonisation remain unassigned.

The gap between diarylpropanol and five-carbon products is not a minor naming issue. Carbon loss must be balanced chemically, and lactone formation may precede or follow further oxidation. Early Eubacterium isolate studies and later cocultures recover plausible intermediates [69,71], but metabolite order in mixed culture cannot distinguish enzyme-catalysed lactonisation from spontaneous cyclisation. Isotope-labelled intermediates and native deletions are still needed.

Human radiotracing shows that five-carbon ring-fission products contribute substantially to absorbed epicatechin-derived material [72]. It does not show whether the carriage of cber, cadh or ecadh predicts an individual’s chiral valerolactone profile. That link requires strain-resolved expression, chiral-metabolite analysis and timed human sampling within one study. Stereoselective enzyme chemistry is a plausible source of variation, not a validated response biomarker.

This matters for foods containing catechin stereoisomers, gallates and oligomeric procyanidins. Their microbial products overlap analytically, but may enter the pathway through different organisms or at different rates. A producer classification based on one pooled PVL measurement may hide substrate-specific bottlenecks visible only through chiral, time-resolved analysis.

### 4.6. Microbial Activation of Glucosinolates

Glucosinolate exposure reflects two competing activation systems. Cutting raw crucifers mixes plant myrosinase with its substrates, whereas heating inactivates the enzyme and shifts conversion towards the lower gut. Raw broccoli yielded much greater and earlier sulforaphane availability than cooked broccoli [73,74]. A crossover trial found high, fairly uniform recovery from preformed sulforaphane but low, variable recovery from glucoraphanin, which first required conversion [75]. With plant myrosinase denatured, late sulforaphane recovery after high-glucoraphanin broccoli still varied markedly between participants [76].

The difference is nutritionally substantial. In one small crossover study, estimated sulforaphane bioavailability was about 37% from raw broccoli and 3.4% from cooked broccoli; median peak time shifted from roughly 1.6 to 6 h [74]. In the Qidong beverage trial, urinary recovery was about 70% from preformed sulforaphane but 5% from glucoraphanin [75]. Though formulation-specific, these figures show that preserved plant enzyme activity can outweigh microbiota-dependent rescue.

Microbial activation was demonstrated before its enzymes were identified. A human Bacteroides thetaiotaomicron strain produced allyl isothiocyanate from sinigrin in monoassociated rats [77]. A dynamic human-colon model showed partial conversion followed by further isothiocyanate metabolism [78]. Together, these studies demonstrated microbial conversion under controlled in vivo and colon-model conditions, but did not identify the responsible catalyst.

In B. thetaiotaomicron, BT2160 regulates the BT2159–BT2156 locus. Native deletions, reconstruction in non-metabolising Bacteroides fragilis and purified-protein assays refute a one-enzyme myrosinase model. BT2157 is required for detectable whole-cell conversion under the tested conditions, whereas BT2158 is required for full activity because its deletion left trace conversion; the complete four-gene module is sufficient to confer the strongest heterologous phenotype. Purified BT2158 combined with BT2156 or BT2157 catalyses conversion [79]. NAD+ enhanced activity, but the redox and glycosidic sequence remains unresolved.

The results differ across experimental levels without being contradictory. A minimal purified combination shows catalytic compatibility, whereas maximal whole-cell conversion also depends on protein stoichiometry, transport and redundant functions. Proposed oxidised or phosphorylated intermediates were not detected, leaving the order of redox assistance and thioglucoside cleavage unresolved. BT2157 should therefore not be described as a conventional bacterial myrosinase.

Substrate disappearance does not establish isothiocyanate formation. Cells carrying the locus produced benzyl isothiocyanate from glucotropaeolin and sulforaphane from glucoraphanin, whereas glucobrassicin was consumed without its expected product [79]. Other gut isolates show species- and side-chain-dependent activity, including glucoraphanin reduction to glucoerucin and erucin formation [80]. Each glucosinolate therefore requires the measurement of its own stable products.

Gnotobiotic mice carrying wild-type B. thetaiotaomicron excreted more sulforaphane conjugates after broccoli than mice carrying an isogenic ΔBT2157 strain [79]. In humans, ex vivo faecal glucoraphanin conversion varies and relates to in vivo isothiocyanate excretion [81]. The BT2159–BT2156 cluster was common in surveyed metagenomes, but only a minority of carriers had detectable transcripts in paired data [79]. Predictive studies should control broccoli processing and measure residual plant myrosinase, microbial expression and matched mercapturic-acid products. Stool DNA alone conflates food preparation with microbial physiology.

Even gene-to-host-product evidence does not establish benefit. The controlled mouse experiment links BT2157 to absorbed sulforaphane-derived conjugates. It neither measures long-term disease outcomes nor shows that this locus dominates conversion in a complex human microbiota. A direct human study would compare repeated myrosinase-standardised meals while measuring locus transcripts and side-chain-specific products in the same participants.

### 4.7. Resveratrol and Hydroxycinnamate Transformations

#### 4.7.1. Resveratrol Reduction

Controlled faecal incubations combined with oral dosing identified dihydroresveratrol as a major microbial product and lunularin-related metabolites in some individuals. Slackia equolifaciens and Adlercreutzia equolifaciens were isolated as reducers before their enzyme was known [82]. Among 195 volunteers, 74% produced lunularin-related products and 26% lacked this downstream phenotype [44]. Resveratrol is absorbed, but free-parent exposure remains very low because conjugation and elimination are rapid [83].

Dihydroresveratrol formation and downstream dehydroxylation are distinct phenotypes. A community may reduce the central double bond yet fail to remove the para hydroxyl group needed for lunularin formation. The 195-person classification thus localises variation to the downstream branch more precisely than a binary resveratrol-metaboliser label, although the responsible dehydroxylase and its population distribution remain unknown [84].

Two studies independently assigned the alkene reduction. In E. lenta DSM 2243, the resveratrol-induced Elen_288 is required for dihydroresveratrol formation because deletion abolished it; the transfer of the Elen_288–Elen_289 reductase–regulator cassette is sufficient to confer activity on a non-metaboliser. Polydatin deglycosylation was common across isolates, but resveratrol reduction was strain-restricted [85].

Resveratrol inhibited susceptible gut isolates from 125 µM, whereas dihydroresveratrol showed little inhibition up to 1 mM. Wild-type E. lenta, unlike the reductase mutant, protected defined communities; gnotobiotic models also shifted towards dihydroresveratrol-derived conjugates when the reducer was present [85]. This demonstrates local detoxification under the tested conditions, not clinical benefit in humans.

In E. lenta J01, purified FAD-containing RER catalyses the reduction in the same double bond, and heterologous expression is sufficient to confer activity. Combining resveratrol with a producer or engineered bacterium increased product formation and improved outcomes in acute mouse colitis [86]. The 100 mg kg^−1^ dose and engineered delivery represent microbiome pharmacology, not ordinary dietary exposure.

The reported J01 RER sequence shares 556 of 557 residues with DSM 2243 Elen_0288 (Elen_288 in the deletion study; ACV54279.1). Thus, deletion/cassette evidence and purified-protein evidence both concern near-identical variants of one conserved reductase locus [85,86].

#### 4.7.2. Hydroxycinnamate-Derived Hydrocaffeic Acid

Hydroxycinnamates reach the colon as free acids, food-matrix esters and products released from larger polyphenols. Nine human donor communities differed in the order of chlorogenic-acid ester hydrolysis, hydrogenation and dehydroxylation, yet converged on 3-(3-hydroxyphenyl)propanoic acid [87]. The Bifidobacterium animalis feruloyl esterase Balat_0669 catalyses initial chlorogenic-acid hydrolysis [88].

Downstream reactions can converge on hydrocaffeic acid (HCA; 3-(3,4-dihydroxyphenyl)propionic acid). Metatranscriptomics identified an HCA-induced locus in Gordonibacter pamelaeae. Its two-subunit bis-molybdopterin-guanine-dinucleotide enzyme, Gp Hcdh, catalyses para-dehydroxylation to form 3-(3-hydroxyphenyl)propionic acid. Activity-guided purification and product-specific kinetics support this assignment [89].

The corresponding hcdh locus in Gordonibacter urolithinfaciens is required for HCA dehydroxylation and the associated rise in cellular ATP, because deletion abolished both, consistent with anaerobic energy conservation. The gene was also strongly expressed relative to candidate catechol dehydroxylases in two human metatranscriptomic datasets [89], although those datasets do not establish population prevalence.

The human finding remains observational. Among participants with low Gp Hcdh expression, greater cruciferous-vegetable intake was associated with lower C-reactive protein; no such association was observed among those with high expression, with evidence of interaction. Neither HCA nor its dehydroxylated product was measured; other inflammatory markers did not show the same pattern, and diet was not randomised. The data therefore suggest that microbial HCA respiration may modify exposure to a food-derived catechol, but cannot determine whether the substrate, product, organism or correlated dietary pattern caused the CRP difference [89]. A mechanistic human study should combine a characterised hydroxycinnamate source with time-resolved measurements of HCA and 3-(3-hydroxyphenyl)propionic acid and concurrent hcdh transcription rather than gene abundance alone. Table 1 records the strongest direct evidence and where inference begins.

## 5. High-Exposure Pathways with Incomplete Enzymology

Some of the products most abundant after phytochemical intake arise from pathways whose human exposure is better defined than their microbial enzymology. Three questions must be kept separate: how much dietary precursor reaches the colon, which transformations faecal communities or isolates perform, and how much of the resulting product contributes to circulating or urinary exposure.

### 5.1. Flavan-3-Ols to Phenyl-γ-Valerolactones

Phenyl-γ-valerolactones (PVLs) combine high human exposure with a partially resolved microbial pathway. After oral administration of [2−^14^C]-(−)-epicatechin, total recovery reached 95 ± 1% in urine and faeces. Structurally related metabolites formed predominantly in the proximal intestine accounted for about 20% of the dose; approximately 42% appeared as five-carbon ring-fission metabolites, including PVL and hydroxyphenylvaleric-acid derivatives, and 28% as smaller phenolic and hippuric-acid derivatives attributed to lower-intestinal catabolism [72]. PVL conjugates appeared later than proximal epicatechin conjugates, reached summed submicromolar plasma concentrations and remained detectable at 24 h. Free epicatechin was not detected. The microbial branch therefore accounts for much of the absorbed labelled dose.

Metabolite studies support a distributed sequence. Human faecal bacteria produced ring-opened and dehydroxylated products from epicatechin gallate [90]. Eggerthella lenta rK3 opened the flavan-3-ol C-ring; Flavonifractor plautii aK2 produced a phenyl-γ-valerolactone (PVL) and hydroxyphenylvaleric acid from this intermediate [71]. Related isolates differed by stereoisomer and dependence on hydrogen or formate [91]. Human faecal communities produced distinct profiles from catechin, epicatechin, A- and B-type dimers and food procyanidins [92]. These studies establish organism- and substrate-dependent chemistry, but not enzymes for carbon loss, lactonisation and terminal dehydroxylations.

Faecal conversion rates and product distributions varied markedly among 24 donors [93]. Green-tea studies recovered substantial delayed valerolactone and phenolic-acid output; 48 h urine collection captured products missed by shorter sampling [94,95,96]. The three proposed urinary response clusters after repeated green-tea and green-coffee intake were derived from only 11 completers and remain exploratory [97]. In a larger validation with authentic standards, one PVL sulfate–glucuronide pair tracked flavan-3-ol intake but retained approximately 60% interindividual variation [98]. A “PVL metabotype” is therefore meaningful only when the dose, analytes and collection interval are explicit.

The ileostomy–colon-intact comparison is particularly informative. Early flavan-3-ol conjugates accounted for a modest fraction of intake, whereas urinary phenolic catabolites approached 40% in participants with a colon [96]. This independently supports a large distal contribution, although non-specific smaller acids cannot all be attributed uniquely to tea. Repeated collections matter: an apparent “low converter” at 8–12 h may be a slow converter sampled before the PVL peak.

The mechanistic gap is clear. Humans are exposed to PVL conjugates and selected bacteria reproduce early and middle reactions, but enzymes for C-ring opening, carbon loss, lactonisation and many terminal dehydroxylations remain largely unassigned. A major PVL increased Nrf2-regulated gene expression in cells, making these metabolites biologically interesting; this does not show that they mediate clinical effects of flavan-3-ol-rich foods [99].

Closing this gap requires labelled, chiral intermediates, not only the parent flavan-3-ol. Activity-guided isolation could then distinguish an enzyme that generates the lactone from one that consumes a preceding diarylpropanol. Measuring microbial transcription and native PVL conjugates in the same participants would test whether the newly assigned early genes explain substantial human flux.

### 5.2. Anthocyanins to Phenolic Acids

Anthocyanins pose a different attribution problem because microbial metabolism overlaps with intrinsic chemical instability. In a ^13^C-cyanidin-3-glucoside study, eight men received 500 mg. Estimated relative bioavailability from urine plus breath was 12.38 ± 1.38%, while total label recovery across urine, breath and faeces ranged from 15.1% to 99.3%. Labelled metabolites reached a mean plasma maximum near 6 μM at approximately 10 h; several had 12–52 h half-lives [100]. Protocatechuic acid (PCA) was a major product after blood-orange juice [101], and targeted profiling detected many more phenolic metabolites than intact anthocyanin conjugates [102]. Parent-only assays underestimate exposure but do not identify the pigment-cleavage catalyst.

At near-neutral intestinal pH, anthocyanin aglycones can open and fragment without a dedicated bacterial enzyme. Human faecal communities and selected isolates accelerate cyanidin-3-glucoside loss and produce PCA and phloroglucinol-related products [103]. Yet several products also formed in germ-free rats, usually at lower abundance; only some depended on microbiota colonisation [104]. Detection in germ-free animals is not a purely chemical blank: host β-glucosidases may contribute to deglycosylation, while enterocytes and liver can conjugate and further oxidise anthocyanin-derived intermediates. Attribution is therefore tripartite—spontaneous chemistry, microbial catalysis and host metabolism—rather than binary. That experiment addressed product formation in animals and accompanying in vitro systems, not human health effects. β-Glucosidase activity can explain deglycosylation by isolates, but most studies neither identify the responsible enzyme sequence nor close the carbon balance from aglycone to each aromatic fragment.

This mixed origin has practical consequences. Sterile incubations must match the pH, oxygen and matrix of faecal cultures; otherwise, chemical cleavage may be attributed to bacteria. Faster disappearance in faecal culture is insufficient unless product recovery exceeds the sterile background. Position-specific labels across both aromatic rings could show whether proposed PCA and phloroglucinol products close the same carbon balance.

Future faecal-incubation studies should report anthocyanin structure, purity and starting concentration; matrix or simulated-digestion history; donor number and pooling; stool collection-to-inoculation time, storage and inoculum density; medium composition; pH at every sampling point; temperature, light exposure, headspace composition and redox state; and sampling and quenching times. Each time course should include matched substrate-in-medium, no-substrate faecal and non-viable-inoculum controls. Parent disappearance should be accompanied by authentic-standard product recovery and, where feasible, position-specific isotope balance; microbial formation should be reported as net product above the time-matched abiotic background rather than as accelerated parent loss alone.

PCA, vanillic acid, ferulic acid and hippuric acid are non-specific endpoints derived from other polyphenols, foods and endogenous aromatic metabolism. A labelled precursor can trace a carbon atom to the administered anthocyanin, but without additional controls cannot distinguish spontaneous cleavage from bacterial or host catalysis. The main uncertainty is therefore quantitative: how much of each phenolic product arises through chemistry, microbial enzymes or subsequent host processing?

Using these acids as unqualified anthocyanin biomarkers risks source misclassification. Labelled carbon, diagnostic conjugation patterns and a timed dietary challenge provide stronger evidence than concentration alone. The issue is quantitative attribution, not whether phenolic catabolites exist.

### 5.3. Stilbenes and Curcuminoids

Human resveratrol exposure is dominated by host conjugation rather than the microbial branch. In a radiotracer study, at least 70% of a 25 mg oral dose was absorbed, yet free resveratrol remained below 5 ng/mL; sulfate and glucuronide metabolites dominated systemic exposure [83]. Dihydroresveratrol, lunularin and 3,4′-dihydroxy-trans-stilbene were detected after intake, but the profiles that varied markedly between individuals and the downstream dehydroxylating organisms remain unidentified [82].

Enzyme assignment has closed only the first step. A screen of taxonomically diverse gut bacteria identified respiratory-like reductases that use dietary and host metabolites, including resveratrol, as electron acceptors [105]. A resveratrol ene-reductase was subsequently identified as the initiating catalyst [86]. Its abundance and expression after dietary dosing, the downstream dehydroxylating organisms and each microbial product’s contribution to human plasma exposure remain unresolved.

Curcumin presents the converse: a convincing microbial enzyme is known, but human exposure to its products is poorly quantified. CurA, an NADPH-dependent reductase from intestinal Escherichia coli, catalyses the sequential reduction in curcumin to dihydrocurcumin and tetrahydrocurcumin [106]. A Blautia isolate produces demethylated curcumin and related curcuminoid products, revealing a parallel branch [107]. The prevalence and expression of both activities in human communities remain uncertain, and host tissues can generate similar reduced products. After 10–12 g curcumin, human pharmacokinetic studies mainly detected glucuronide and sulfate conjugates; free curcumin appeared in one participant at one time point [108]. These findings establish extensive host handling, not CurA- or Blautia-derived systemic exposure. Claims that tetrahydrocurcumin or demethylated metabolites explain responses to oral curcumin remain premature because precursor-specific carbon balance and time-resolved native-conjugate measurements are lacking. The principal unresolved mechanistic steps and the experiments required to distinguish among competing pathways are summarized in Table 2.

## 6. Microbial Division of Labour and Interindividual Variation

Variation can arise at each reaction rather than being reducible to one taxonomic node. Lignan formation requires several organisms, and its entry enzyme Ber varies among E. lenta strains [54]. In the urolithin route, terminal products depend on upstream intermediate suppliers, carriage of ucd or uxd and substrate-induced transcription [42,43]. A low-abundance organism may therefore control flux, yet its presence alone may be insufficient without a partner or induced function.

Human phenotypes capture the net result, not its cause. Equol production is relatively persistent and generally not induced by sustained soy intake [33,35]. Urolithin metabotypes A, B and 0 describe qualitative terminal products; proposed PVL clusters are quantitative and highly dose- and sampling-sensitive [97,109]. Product absence may reflect missing genes, weak expression, slow precursor release, rapid consumption, transit or host disposition.

Between-person variability must be separated from within-person instability. A reproducible equol phenotype can coexist with wide quantitative variation in excretion. Likewise, an age-associated cross-sectional distribution of urolithin metabotypes does not show that individuals switch categories over time. Repeated challenges using the same processing and collection protocol are needed before calling a microbial phenotype stable. Antibiotics, habitual diet and recent substrate exposure can then be tested as modifiers.

Taxon–metabolite correlations are discovery tools, not pathway proof. Strong human studies combine a defined substrate challenge and authentic-standard metabolite analysis with strain-resolved gene or transcript measurements in the same participants. This distinction parallels the concept of functional dysbiosis, in which altered microbial metabolic capacity is more informative than taxonomic shifts alone [110]. Here, functional dysbiosis denotes a shift in community-level metabolic capacity or output that may occur with or without a detectable change in taxonomic composition [110]. Urinary output integrates microbial ecology with absorption, conjugation and renal clearance; it cannot be attributed to the microbiota alone.

Interpretation of gnotobiotic evidence depends on community complexity. Monoassociated models examine one strain without competitors, defined consortia test specified cross-feeding, and animals colonised with complex human-derived microbiota retain more donor functions, nutritional competition and spatial interactions. These designs are not interchangeable: single-strain and defined-community models maximise mechanistic attribution but simplify ecology, whereas human-derived communities undergo selective engraftment and remain constrained by a nonhuman host. We therefore extrapolate gnotobiotic findings to human physiology only when they agree with human substrate-to-product measurements [111].

Variation in food preparation and composition can rival variation in the microbiota. Milling changes lignan availability, heating alters plant-myrosinase activity, and glycoside form changes proximal isoflavone kinetics. Without characterising these inputs, a metabotype study may misattribute processing variability to microbial ecology. A standardised precursor challenge, however, can reveal microbial flux when ordinary meals provide too little substrate for the phenotype to be expressed.

## 7. From Colonic Product to Circulating and Urinary Metabolite Exposure

A faecal-culture product becomes a human exposure only if it forms before further microbial consumption, crosses the epithelium and survives host metabolism until sampling. Sulfation, glucuronidation and methylation mean that blood usually contains species unlike those tested in culture. Precursor-specific tracers provide the strongest carbon balance: labelled epicatechin showed major lower-intestinal output [72], whereas labelled cyanidin-3-glucoside produced delayed, persistent metabolites with wide interindividual recovery [100]. A tracer establishes precursor origin more readily than it identifies the responsible enzyme or organism.

The downstream effects of microbiota-derived metabolites therefore depend not only on their chemical identity but also on their balance and bioavailability [112]. Urine integrates formation, absorption, conjugation and renal clearance; faecal abundance may indicate rapid production, poor absorption or failed downstream consumption. In human coffee studies, dihydrocaffeic and dihydroferulic conjugates appeared after the early absorption phase and represented a substantial share of urinary hydroxycinnamate products [113,114]. Their timing supports colonic formation but does not identify Hcdh or another catalyst. Paired faecal and human detection of resveratrol products is stronger than either alone, although the route to each circulating conjugate remains incomplete [82].

Direct-metabolite dosing asks a different pharmacokinetic question. Stable-isotope-labelled S- and R-equol were rapidly absorbed, with elimination half-lives of approximately 7–8 h and different systemic availability between enantiomers [115]. This describes host disposition after microbial conversion, not how much daidzein an individual’s microbiota converts. Likewise, uniform plasma exposure after a urolithin A capsule does not imply uniform conversion from ellagitannins.

Analytical choices alter the apparent pathway. With authentic standards, selected PVL sulfate and glucuronide conjugates became useful intake biomarkers; smaller phenolic acids and hippurate have many sources [98]. Enzymatic deconjugation improves sensitivity but erases conjugation position and circulating identity. Standardised names are especially important for polyphenol catabolites and phase-II metabolites [116]. Exact mass and fragment matching without authentic-standard retention-time confirmation warrants a lower identification-confidence level, not an unqualified structure [117].

Sampling design is part of structural identification. Early plasma collections favour proximal conjugates; 24–48 h urine captures slower colonic products but loses concentration–time information. Matched plasma, urine and faecal time courses are rarely feasible at scale, yet one dense pharmacokinetic subset is needed before interpreting a single epidemiological urine sample as pathway flux. When biological identity matters, recovery should be reported for native conjugates and after hydrolysis.

Biological interpretation demands equal discipline. Activity of an unconjugated microbial product at a supraphysiological concentration does not establish the activity of its circulating sulfate or glucuronide. PVL–Nrf2 findings remain cell-culture evidence, not human mediation [99]. Direct dosing of a purified microbial product tests the molecule while bypassing matrix release and microbial production.

Three lines of evidence must agree: precursor-derived carbon in the native human product, the microbial reaction under intestinal conditions and active catalytic machinery in that exposure context. PVLs have strong mass balance but incomplete enzyme assignment; anthocyanin phenolic acids have labelled exposure but mixed origins; resveratrol and curcumin have selected enzymes without a complete microbial-product balance in humans. These are distinct gaps and should not be collapsed into a single claim of “microbiome-mediated bioavailability”.

Table 3 compares what human studies actually measured with the narrower interpretation that those measurements support.

## 8. Translational Interpretation at Physiologically Plausible Exposure

Biological relevance requires the right molecule in the right compartment. A food label may name a transient luminal substrate, while peripheral tissues encounter microbial products and host sulfates or glucuronides. Low faecal abundance may instead reflect efficient absorption. Oral dose, plasma concentration and colonic exposure are not interchangeable. Across natural bioactives, pharmacokinetic variability, microbiota-dependent biotransformation and source standardisation remain major barriers to clinical translation [110].

### 8.1. Local Colonic Tissue Exposure Is Distinct from Systemic Circulation

Resveratrol makes this distinction explicit. Free-parent exposure remains low even after gram-range dosing, whereas sulfate and glucuronide metabolites reach much higher concentrations [83,119]. Resveratrol and five conjugates are nevertheless detectable in colorectal tissue after repeated dosing, despite near-undetectable free parent in plasma [120]. This supports local tissue exposure, not the general exposure of distant organs. The overlap between faecal resveratrol concentrations and bacterial inhibitory thresholds supports community detoxification in donor-derived mouse systems [85], but not a human systemic anti-inflammatory mechanism.

Curcumin shows a similar compartmental split. After patients with colorectal cancer received 3.6 g of curcumin daily, low nanomole-per-gram concentrations were detected in colorectal tissue while plasma exposure remained minimal; pharmacodynamic endpoints were inconsistent [121]. Local exposure can therefore be real without proving efficacy or a microbial source. By contrast, epicatechin radiotracing shows the delayed circulation of conjugated five-carbon products without detectable unchanged parent [72]. Systemic models should use those native forms and time courses, not arbitrarily high parent aglycone.

These compartments require different exposure metrics: faecal-water concentration and residence time for luminal effects, tissue concentration for epithelial exposure, and native-conjugate plasma area under the curve for systemic exposure. Detection in faeces or colorectal tissue cannot be extrapolated to systemic exposure, whereas low plasma concentrations do not exclude a local colonic effect.

### 8.2. Human Phenotype Evidence Remains Uneven

Urolithins best illustrate the difference between dietary formation and direct-product dosing. In a small raspberry crossover trial, plasma urolithin conjugates correlated with improvement in flow-mediated dilation, but the small sample and correlational analysis do not establish mediation [122]. Direct urolithin A dosing produced molecular signatures compatible with better mitochondrial function [123]. In a later randomised trial, however, urolithin A did not improve the primary six-minute-walk or maximal-ATP outcomes, despite changes in secondary endurance measures and biomarkers [124]. These direct-dosing trials test administered urolithin A, not ellagitannin-rich foods or a producer metabotype.

Primary and secondary outcomes must remain distinct. Biomarker changes can support target engagement but do not override a null prespecified functional endpoint. Conversely, a null capsule trial would not exclude the local intestinal effects of ellagitannins or another urolithin mixture. Precursor foods, producer metabotypes and purified metabolites are related but non-equivalent interventions.

For glucosinolates, bacterial deletion and gnotobiotic feeding link a microbial locus to host-accessible sulforaphane conjugates, while human feeding shows strong processing effects and variable lower-gut conversion [75,79]. No study yet combines microbial transcription, sulforaphane kinetics and a prespecified human health endpoint. Resveratrol and HCA fall further short: resveratrol has strong reductase genetics but no demonstrated human benefit of the metabotype; HCA has a causal bacterial energy-conservation mechanism but only an observational diet–enzyme-expression interaction [85,89].

This evidence order prevents reverse inference. A favourable clinical association after broccoli does not identify BT2159–BT2156 as its mediator; nor does a bacterial resveratrol-reductase knockout show that dihydroresveratrol explains a human supplement response. The missing bridge is the concurrent measurement of precursor, pathway activity, native product and endpoint, ideally with the selective perturbation of the microbial reaction.

### 8.3. Choosing Chemical Form, Concentration and Endpoint

Studies intended to explain dietary effects should use the forms and concentrations measured in vivo, a long-standing principle still often ignored [125]. Plasma data for a native conjugate suit systemic models; faecal water, ileal effluent or validated gastrointestinal simulations better represent microbial exposure. Protein and matrix binding determine free concentration. Delayed microbial products may also require pulse or repeated-dose designs rather than constant exposure at the measured maximum.

Mixtures should reflect measured molar proportions, not convenient equal concentrations. If authentic conjugates are unavailable, aglycone testing may be exploratory, but the mismatch in chemical form must be stated and the concentration range should include estimated human exposure. A negative result under those conditions can exclude an attractive direct mechanism while leaving tissue deconjugation or local luminal effects as separate hypotheses.

Endpoints should match the demonstrated chemistry. Community growth and substrate-to-product balance suit microbial detoxification; receptor engagement at measured free concentrations better tests host signalling. A generic antioxidant assay using tens or hundreds of micromolar parent aglycone neither identifies the active in vivo species nor distinguishes direct scavenging from adaptive signalling. Negative results at plausible exposure are informative. Reaction-level evidence is often strong, but any improvement in human health remains a separate claim requiring matched exposure and phenotype data.

## 9. Conclusions

Few phytonutrient transformations are resolved from dietary substrate through microbial catalyst to a product measured in humans. The strongest examples have different architectures: a strain-variable equol cluster, a distributed enterolignan consortium, inducible urolithin branches, stereoselective flavonoid enzymes and a multicomponent glucosinolate-activating locus. Resveratrol and hydrocaffeic acid also show that microbial metabolism can protect neighbouring bacteria or remove a functional group, rather than simply “increase bioactivity”.

Pathway flux depends on precursor delivery, strain-level gene content, transcription, cofactors, partner organisms, residence time, product consumption, absorption and host conjugation. Taxonomic or gene detection cannot replace a controlled substrate challenge with targeted metabolite measurement. Human studies should pair strain-resolved genomic and transcriptomic data with measurements of native substrates, intermediates and host-conjugated products.

Major gaps remain despite high human exposure. Phenyl-γ-valerolactones and anthocyanin-derived phenolic acids account for substantial dose-related material, yet several enzymes and the relative contributions of spontaneous, microbial and host chemistry remain unknown. Leaving these arrows unresolved is more informative than filling them computationally. Biological relevance should be tested using forms, concentrations and anatomical sites demonstrated in humans. This reaction-centred, exposure-aware approach provides a firmer basis for explaining interindividual responses to phytonutrient-rich foods.

## Figures and Tables

**Table 1:** Reaction-level resolution of representative gut microbial phytonutrient pathways.

Dietary Precursor → Microbial Product.	Assigned Reaction and Catalyst	Organism (s)	Strongest Direct Evidence	Current Boundary
Daidzein → S-equol	DZNR reduction; DDRC racemisation; DHDR and THDR reductions	*Slackia* sp. NATTS; *Lactococcus* sp. 20–92; *Adlercreutzia equolifaciens*	Purified/recombinant enzymes and heterologous four-gene reconstruction [18,19,23]	Native-host genetics and person-level pathway flux remain incomplete.
Ellagic acid → urolithin A, isourolithin A and urolithin B	Early dehydroxylations; UcdCFO 9-dehydroxylation; UxdFOC 10-dehydroxylation	*Gordonibacter*, *Ellagibacter* and selected *Enterocloster* strains	Isolates, heterologous enzyme complexes, defined consortia and expression-linked faecal conversion [40,42,43]	The lactone-opening and earliest dehydroxylating enzymes are unresolved.
Pinoresinol → enterolactone	Ber benzyl-ether reductions; Glm-associated O-demethylation; Cldh-associated dehydroxylation; Edl oxidation	*Eggerthella lenta*, *Blautia producta*, *Gordonibacter pamelaeae*, *Lactonifactor longoviformis*	Ber/Edl heterologous activity, four-isolate consortium and gnotobiotic validation [54]	Glm and Cldh have not been independently reconstituted as purified catalysts.
Flavones/flavonols → dihydrochalcones and phenylpropionates	FLR C2=C3 reduction; Fcr ring opening; Phy C–C hydrolysis; chalcone-isomerase branch	*Flavonifractor plautii*; *Eubacterium ramulus*	Purified enzymes; *flr* knockout/complementation in *Clostridium ljungdahlii* [60,64,65]	Electron delivery to FLR and quantitative continuity of the route in humans are unknown.
Catechin/epicatechin → ring-opened and hydroxyvalerate products	Cber ether cleavage; enantiocomplementary Cadh/eCadh dehydroxylation; Vadh reactions	*E. lenta*, *Gordonibacter* spp. and *F. plautii*	Heterologous reconstruction, purified enzymes and cocultures [70]	A-ring carbon loss and phenyl-γ-valerolactone formation are not fully assigned.
Glucoraphanin → sulforaphane	Multicomponent BT2159–BT2156 pathway; BT2158 with BT2156 or BT2157 is biochemically active	*Bacteroides thetaiotaomicron*	Native deletions, heterologous reconstruction, purified combinations and gnotobiotic feeding [79]	Detailed redox/glycosidic sequence and side-chain-specific product fate remain open.
Resveratrol → dihydroresveratrol	Alkene reduction by the near-identical Elen_0288/RER orthologues	Strain-specific *E. lenta* isolates; other Coriobacteriia	Native loss/gain of function and purified or heterologously expressed reductase [85,86]	Population prevalence, in-colon expression and downstream dehydroxylations are incomplete.
Hydrocaffeic acid → 3-(3-hydroxyphenyl)propionic acid	Gp Hcdh para-dehydroxylation coupled to anaerobic energy conservation	*Gordonibacter pamelaeae* and *G. urolithinfaciens*	Activity-guided purification, targeted deletion and human metatranscriptomics [89]	Precursor-specific human metabolite exposure and causal host effects are untested.

Abbreviations: Ber, benzyl-ether reductase; Cadh, (+)-catechin dehydroxylase; Cber, catechin benzyl-ether reductase; Cldh, catechol lignan dehydroxylase; DDRC, dihydrodaidzein racemase; DHDR, dihydrodaidzein reductase; DZNR, daidzein reductase; eCadh, (−)-epicatechin dehydroxylase; Edl, enterodiol-lactonizing enzyme; Fcr, flavanone- and flavanonol-cleaving reductase; FLR, flavone reductase; Glm, guaiacol lignan methyltransferase; Gp Hcdh, Gordonibacter pamelaeae hydrocaffeic acid dehydroxylase; Phy, phloretin hydrolase; RER, resveratrol reductase; THDR, tetrahydrodaidzein reductase; UcdCFO, urolithin C dehydroxylase complex; UxdFOC, urolithin 10-position (X) dehydroxylase complex; Vadh, valeric acid dehydroxylase acting on catechin-derived phenolic acids.

**Table 2:** Translates the main mechanistic gaps into experiments that distinguish catalytic pathways from taxonomic associations or metabolite co-occurrence.

Pathway	Established Observation	Exact Unresolved Step	Discriminating Experiment
Flavan-3-ols → phenyl-γ-valerolactones	Human mass balance and early stereoselective bacterial reactions [70,72]	A-ring carbon loss, lactonisation and several terminal dehydroxylations	Activity-guided anaerobic fractionation with isotope-labelled intermediates, followed by gene deletion and same-participant transcript–chiral-metabolite testing.
Anthocyanins → phenolic acids	Labelled human products and conversion by faecal communities or isolates [100,103]	Relative shares of spontaneous cleavage, bacterial catalysis and host processing	Parallel sterile, isolate, defined-community and colonised-host experiments using position-specific labels and native-conjugate LC–MS.
Ellagic acid → early urolithins	Producer isolates and resolved terminal Ucd/Uxd dehydroxylases [40,42]	Lactone opening and the earliest dehydroxylations	Substrate-induced proteomics in Gordonibacter with fractionated activity, knockout/complementation and authentic intermediate standards.
Lignans → enterodiol	Defined four-member pathway; Ber and Edl are sufficient in heterologous hosts [54]	Independent catalytic proof for Glm-associated demethylation and Cldh-associated dehydroxylation	Reconstitute full protein/cofactor modules, then test targeted deletions in the native isolates and rescue the consortium phenotype.
Resveratrol → downstream stilbenes	The first alkene reduction is genetically and biochemically assigned [85,86]	Organisms and enzymes producing lunularin and 3,4′-dihydroxystilbene	Feed labelled dihydroresveratrol to responder communities, combine differential metatranscriptomics with isolate recovery, and verify candidate deletions.
Curcumin → reduced and demethylated products	CurA reduction and a separate Blautia demethylation branch are reproduced in isolates [106,107]	Their quantitative contribution to circulating human metabolites	Use oral stable-isotope curcumin with native-conjugate plasma/urine profiling and simultaneous measurement of pathway expression in stool.

Abbreviations: Ber, benzyl-ether reductase; Cldh, catechol lignan dehydroxylase; CurA, NADPH-dependent curcumin/dihydrocurcumin reductase; Edl, enterodiol-lactonizing enzyme; Glm, guaiacol lignan methyltransferase; LC–MS, liquid chromatography–mass spectrometry; Ucd, urolithin C dehydroxylase; Uxd, urolithin 10-position (X) dehydroxylase.

**Table 3:** What human studies establish about microbial phytonutrient exposure.

Precursor/Output	Human Evidence	Measured Output	Variation or Timing	Defensible Interpretation
Soy isoflavones/S-equol	Standardised daidzein or soy challenges with urinary/plasma phenotyping	Chiral S-equol	Producer status differs among populations and is often concordant over years [32,33]	A genuine microbial phenotype; producer status does not guarantee a clinical response.
Ellagitannins/urolithins	Multiple food interventions and direct-product dosing	Urolithin conjugates and faecal terminal products	Cross-sectional metabotype distribution differs with age; direct urolithin A gives more uniform exposure [45,50]	Dietary conversion and direct-metabolite supplementation are distinct interventions.
Flax lignans/enterolignans	Secoisolariciresinol diglucoside pharmacokinetics and crossover intervention	Enterodiol and enterolactone conjugates	Delayed sequential appearance and large between-person excretion differences [58,118]	Systemic exposure is demonstrated; health mediation remains uncertain. Analytical hurdle: deconjugation collapses native conjugate profiles; report native and hydrolysed data separately.
(−)-Epicatechin/five-carbon products	Oral ^14^C mass-balance study	Phenyl-γ-valerolactone and hydroxyvalerate conjugates	About 42% of dose as five-carbon products; later peak than proximal metabolites [72]	Large lower-intestinal flux is established, but the complete enzyme sequence is not. Analytical hurdle: radiolabel recovery cannot resolve PVL regio-/stereoisomers or positional conjugates; use authentic standards, conjugate-specific chromatography and chiral separation.
Cyanidin-3-glucoside/phenolic acids	Oral ^13^C tracer and targeted interventions	Labelled phenolic metabolites, including protocatechuic-acid-related products	Late, persistent exposure and very wide carbon recovery [100,102]	Precursor origin is supported; spontaneous, microbial and host cleavage are not fully separable.
Broccoli glucosinolates/isothiocyanates	Fresh-versus-steamed feeding and paired faecal conversion	Mercapturic-acid isothiocyanate products	Active plant myrosinase increases availability; faecal conversion varies [73,81]	Food processing and microbial activity jointly determine exposure.
Resveratrol/dihydroresveratrol	Controlled intake with paired faecal incubations	Dihydroresveratrol, lunularin and 3,4′-dihydroxystilbene-related products	Qualitatively different product profiles among individuals [82]	Human metabotypes exist; benefit from the reductive phenotype is not established.
Curcumin/reduced or demethylated products	High-dose pharmacokinetic studies	Host glucuronides and sulfates dominate	Microbial-source products are not resolved quantitatively [108]	Known bacterial enzymes cannot yet be assigned a material share of human exposure.

Isotopic notation: ^13^C, carbon-13; ^14^C, carbon-14.

## Data Availability

No new data were created or analysed in this study. Data sharing is not applicable to this article.

## Supplementary Materials

The following supporting information can be downloaded at https://www.mdpi.com/article/10.3390/molecules31162895/s1, Figure S1: Evidence-identification, appraisal, and synthesisworkflow used in this critical narrative review.

## Abbreviations

The following abbreviations are used in this manuscript:

Ber	Benzyl ether reductase
Cadh	(+)-Catechin dehydroxylase
Cber	Catechin benzyl-ether reductase
Cldh	Catechol lignan dehydroxylase
CurA	Curcumin reductase
DDRC	Dihydrodaidzein racemase
DHD	Dihydrodaidzein
DHDR	Dihydrodaidzein reductase
DZNR	Daidzein reductase
eCadh	(−)-Epicatechin dehydroxylase
Edl	Enterodiol-lactonizing enzyme
Fcr	Flavanone- and flavanonol-cleaving reductase
FLR	Flavone reductase
Glm	Guaiacol lignan methyltransferase
HCA	Hydrocaffeic acid
Hcdh	Hydrocaffeic acid dehydroxylase
PCA	Protocatechuic acid
PVL	Phenyl-γ-valerolactone
RER	Resveratrol ene-reductase
SDG	Secoisolariciresinol diglucoside
THDR	Tetrahydrodaidzein reductase
Ucd	Urolithin C 9-dehydroxylase
Vadh	5-(3′,4′-Dihydroxyphenyl)valeric acid dehydroxylase
