# Supplementary material for: How Gut Microbes Transform Dietary Phytonutrients: Enzymatic Pathways and Human Metabolite Exposure
Source: Molecules. 2026 Aug 19;31(16):2895. doi: 10.3390/molecules31162895 (PMC13515148; doi:10.3390/molecules31162895)
Supplement: Supplementary file 1 [file molecules-31-02895-s001.zip › molecules-4497301-supplementary.pdf]

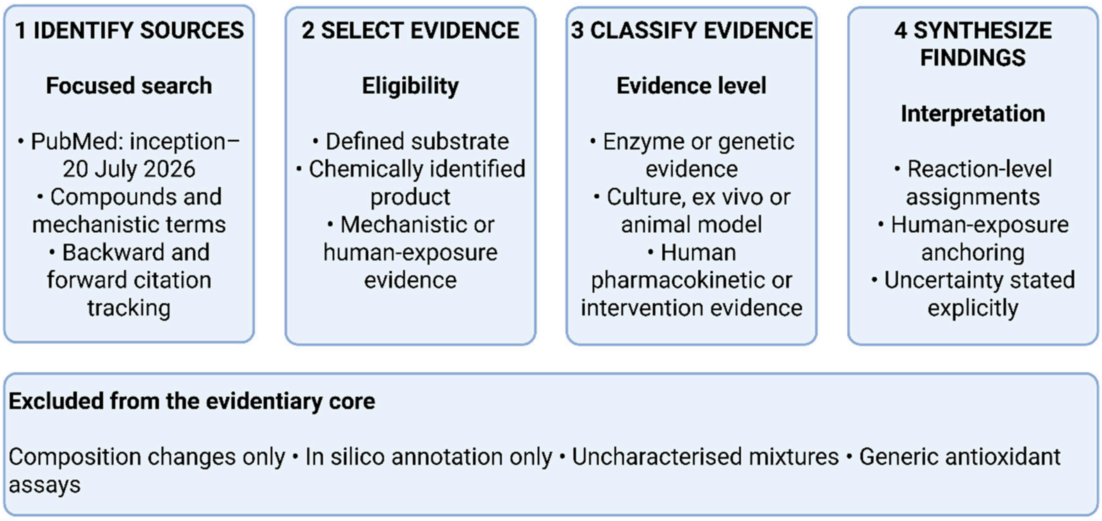

Supplementary Figure S1. Evidence-identification, appraisal, and synthesis workflow used in this critical narrative review. Focused PubMed searches from database inception through 20 July 2026 were supplemented by backward and forward citation tracking. Evidence was retained when it linked a defined substrate to a chemically identified product through mechanistic or human-exposure data and was subsequently classified according to evidentiary level before pathway-level synthesis. Reports limited to compositional changes, in silico annotations, uncharacterised mixtures, or generic antioxidant assays were excluded from the evidentiary core. Created in BioRender. Kumric, M. (2026) https://BioRender.com/m1mogis.
